# Supplementary material for: Identification of a Novel Two-Peptide Lantibiotic from Vagococcus fluvialis
Source: Microbiol Spectr. 2022 Jun 22;10(4):e00954-22. doi: 10.1128/spectrum.00954-22 (PMC9431498; doi:10.1128/spectrum.00954-22)
Supplement: Supplemental file 1 — Table S1 and Fig. S1. Download spectrum.00954-22-s0001.pdf, PDF file, 0.1 MB [file spectrum.00954-22-s0001.pdf]

Supplementary tables and figures.

Table S1 Antibiotic resistance of *Enterococcus faecium* LMG 20705.

Antibiotic	Gene[a]	Gene product	Accession number)
ampicillin[b]	-	-	-
aminoglycoside	*aac*(6')-Ii	aminoglycoside 6'-N-acetyltransferase	WP_002293989.1
clindamycin[b]	*lnu*B	lincosamide nucleotidyltransferase	WP_002294514.1
erythromycin[b]	*erm*B	rRNA adenine N-6-methyltransferase	WP_001038795.1
kanamycin[b]	*aph*(3')-IIIa	aminoglycoside O-phosphotransferase	WP_001096887.1
pleuromutilin	*eat*A	ABC-F type ribosomal protection protein	WP_002296175.1
spectinomycin	*ant*(9)-Ia	aminoglycoside nucleotidyltransferase	WP_002294509.1
streptogramin A[b,*]	*lsa*E	ABC-F type ribosomal protection protein	WP_002294513.1
streptogramin B[b,*]	*msr*C	ABC-F type ribosomal protection protein	WP_063854349.1
streptomycin[b]	*ant*(6)-Ia	aminoglycoside nucleotidyltransferase	WP_001255866.1
streptothricin	*sat*4	streptothricin N-acetyltransferase	WP_000627290.1
tetracycline[b]	*tet*L	tetracycline efflux MFS transporter	WP_002294500.1
	*tet*M	tetracycline resistance ribosomal protection protein	WP_063856394.1
vancomycin[b]/teicoplanin[b]	*van*A	D-alanine-(R)-lactate ligase	WP_001079845.1
	*van*HA	D-lactate dehydrogenase	WP_001059542.1
	*van*RA	DNA-binding response regulator	WP_001280781.1
	*van*SA	histidine kinase	WP_002305818.1
	*van*XA	D-Ala-D-Ala dipeptidase	WP_000402348.1
	vanYA	D-Ala-D-Ala carboxypeptidase	WP_001812592.1
	vanZA	glycopeptide resistance protein	WP_000516404.1

[a] Found in *E. faecium* LMG 20705 genome with AMRFinderPlus
[b] Tested and confirmed by disc diffusion method according to EUCAST
[*] Quinopristin/dalfopristin resistance

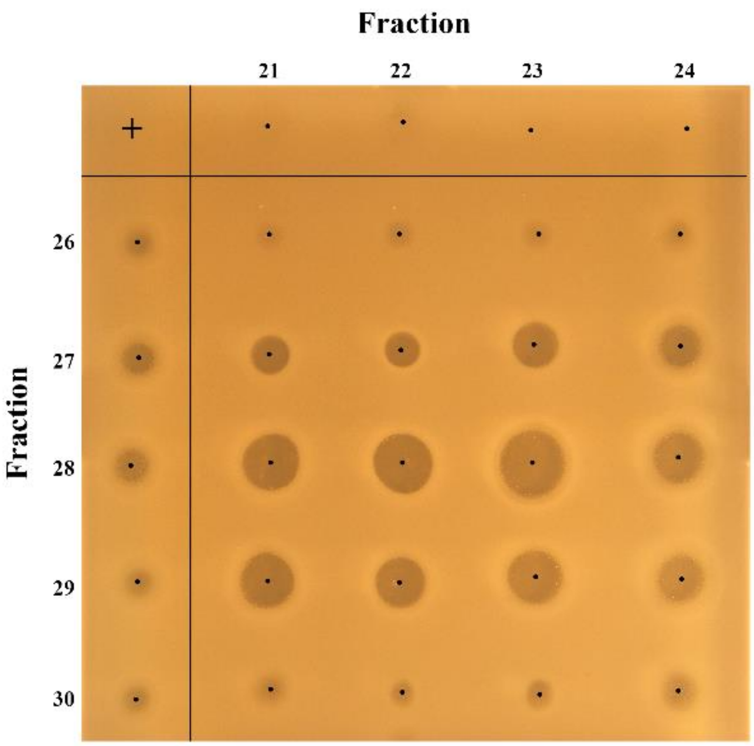

Figure S1 Fractions (1 μl) from reversed-phase chromatography corresponding to the first (21 to 24) and second peak (26 to 30) were spotted individually (to the left and above black bars) and in combination (1:1 v/v ratio) on a lawn of *E. faecium* LMG 20705. Fractions spotted individually produced no or only small/diffuse inhibition zones, some fractions produced large inhibition zones when spotted in combination with the largest zone produced by a combination of fractions 23 and 28.
